# Roles of NUCKS1 in Diseases: Susceptibility, Potential Biomarker, and Regulatory Mechanisms

**DOI:** 10.1155/2018/7969068

**Published:** 2018-01-29

**Authors:** Pengru Huang, Yujie Cai, Bin Zhao, Lili Cui

**Affiliations:** Guangdong Key Laboratory of Age-Related Cardiac and Cerebral Diseases, Affiliated Hospital of Guangdong Medical University, Zhanjiang, Guangdong, China

## Abstract

Nuclear casein kinase and cyclin-dependent kinase substrate 1 (NUCKS1) is a 27 kD chromosomal, highly conserved, and vertebrate-specific protein. NUCKS1 gene encodes a nuclear protein and the conserved regions of NUCKS1 contain several consensus phosphorylation sites for casein kinase II (CK2) and cyclin-dependent kinases (Cdk) and a basic DNA-binding domain. NUCKS1 is similar to the high mobility group (HMG) family which dominates chromatin remodeling and regulates gene transcription. Meanwhile, NUCKS1 is a RAD51 associated protein 1 (RAD51AP1) paralog that is significant for homologous recombination (HR) and genome stability and also a transcriptional regulator of the insulin signaling components. NUCKS1 plays an important role in DNA damage response and metabolism, participates in inflammatory immune response, and correlates with microRNA. Although there is still not enough functional information on NUCKS1, evidences suggest that NUCKS1 can be used as the biomarker of several cancers. This review summarizes the latest research on NUCKS1 about its susceptibility in diseases, expression levels, and regulatory mechanisms as well as the possible functions in reference to diseases.

## 1. Introduction

Nuclear casein kinase and cyclin-dependent kinase substrate 1 (NUCKS1), which is located on human chromosome 1q32.1 [1], is a member of the high mobility group (HMG) family [2]; NUCKS1 is widespread in vertebrates and is expressed ubiquitously by almost all human cell types [2–4]. NUCKS1 plays a significant role in modulating chromatin structure [1] and regulates cellular events such as replication, transcription, and chromatin condensation [2]. Remarkably, in contrast to HMG, NUCKS1 is highly phosphorylated at multiple sites [5, 6]. NUCKS1 can be phosphorylated by kinases, such as casein kinase II (CK2) and cyclin-dependent kinase (Cdk) and DNA-activated protein kinase. Notably, NUCKS1 can also be phosphorylated in all phases of the cell cycle and exhibit the mitosis-specific phosphorylation of threonine residues [4, 7], which is implicated in cell growth and proliferation as well as DNA repair [7–10]. Moreover, posttranslational modifications of NUCKS1, which include acetylation, methylation, ubiquitylation, and formylation, mainly occur in interphase [6]. In addition, in vitro experiments have indicated that NUCKS1 can act as a substrate for second messenger-activated kinases [11]. The aforementioned functions of NUCKS1 contribute to the susceptibility, occurrence, and development of several cancers and other diseases and suggest that NUCKS1 could be a potent marker for such diseases [12]. An understanding of additional potential functions of NUCKS1 may provide new insight into novel therapeutic strategies to address the development of these diseases. In this article, we focus on new evidence regarding the associations between NUCKS1 and susceptibility to diseases; NUCKS1 expression levels in diseases; and regulatory mechanisms involving NUCKS1 in the context of diseases.

## 2. Genetic Susceptibilities Associated with NUCKS1

**Figure 1:**
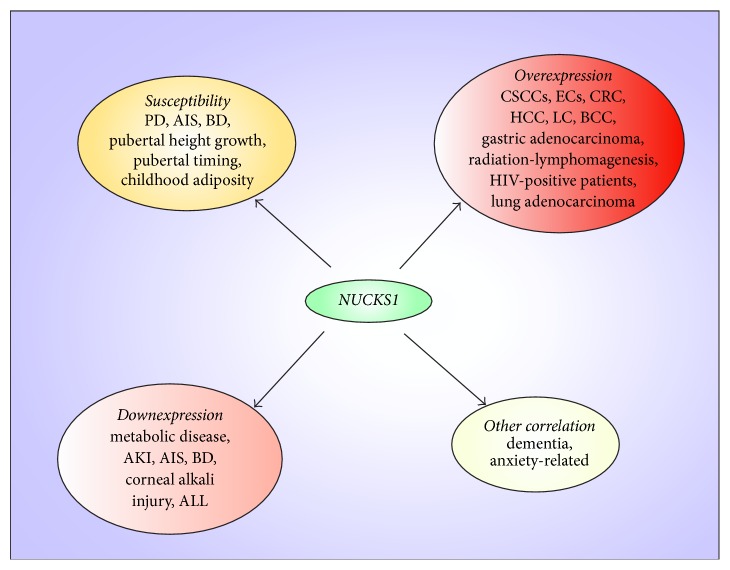
The association between NUCKS1 and diseases/metabolic process. NUCKS1 is one of susceptible genes in Parkinson disease (PD), adolescent idiopathic scoliosis (AIS), bipolar disorder (BD), and so on. NUCKS1 has overexpression in many tumours, such as cervical squamous cell carcinomas (CSCCs), endometrial cancers (ECs), colorectal cancer (CRC), hepatocellular carcinoma (HCC), lung cancer (LC), and basal cell carcinoma (BCC) and in human immunodeficiency virus (HIV) positive patients in in vivo studies. NUCKS1 also has overexpression in lung adenocarcinoma according to in vitro study. But NUCKS1 has downexpression in acute kidney injury (AKI), AIS, BD, and so on, in in vivo studies, as well as in acute lymphoblastic leukemia (ALL) which is in vitro study. Besides, NUCKS1 has correlation with dementia and anxiety.

Increasing evidence support the existence of complex genetic contributions to disease development [13–20]. Genome-wide association studies (GWAS) have shed light on the genetic basis of disease, leading to the identification and replication of risk loci that fit the common disease-common variant hypothesis [13–16, 19]. The NUCKS1 gene, which contains thousands of related single-nucleotide polymorphisms (SNPs), has been reported to be potentially associated with several diseases and with the growth and development of adolescents. GWAS have identified NUCKS1 as one of five transcripts of the PARK16 locus on 1q32, which is a susceptibility locus associated with Parkinson disease (PD) [21]. Furthermore, transcript levels of NUCKS1 (including rs947211 and rs823114) have been shown to be strongly associated with PD in a Japanese population [22]. However, no significant associations between rs823114 of NUCKS1 and PD have been observed in Ashkenazi Jewish [23, 24], Chinese [25], and Scandinavian [26] populations. In addition, rs823128 of NUCKS1 is associated with PD at or close to a genome-wide significance level in Asians but not Caucasians (CEU) [27]. However, in other data, there was strong linkage disequilibrium (LD) for rs823128 in NUCKS1 with five SNPs (rs823123, rs823135, rs823136, and rs823142 in RAB7L1 and rs1620334 in NUCKS1) in all ethnic groups but with rs823092 in NUCKS1 only in Asian groups [28]. Moreover, RAB7L1/NUCKS1 has candidate genetic variants that may be associated with sporadic PD in Han Chinese subjects. Furthermore, for RAB7L1/NUCKS1 rs823118, subjects with the CC or CT genotype had lower risks of PD than subjects with the TT genotype [29]. NUCKS1 (rs4880445) is also a potential susceptibility gene for bipolar disorder (BD) that has been identified in youth at high risk for BD via a combined transcriptome and methylome analysis [30]. Remarkably, a recent GWAS showed that a menarche genomic locus, rs951366 of NUCKS1, is highly enriched for variants that more generally regulate pubertal timing [31]. In addition, rs951366 of NUCKS1 has been reported to be a susceptibility gene of adolescent idiopathic scoliosis (AIS). Moreover, patients with a T allele for rs951366 are more vulnerable to AIS as well as late onset of menarche [32]. Intriguingly, in a longitudinal study, rs823094 of NUCKS1 was found to be associated with pubertal height gain [33]. As noted, NUCKS1 has been identified as a potential susceptibility gene for PD, BD, pubertal height gain, pubertal timing, childhood adiposity, and AIS (Figure 1).

## 3. Levels of NUCKS1 in Diseases

NUCKS1 is ubiquitously expressed in all mammalian tissues [3, 4] and has been confirmed to be overexpressed in many cancers, especially malignant neoplasms. Therefore, NUCKS1 can be used as a marker for various cancers [12, 34–46]. In in vivo studies, a high level of NUCKS1 may be associated with tumor progression and recurrence in cervical squamous cell carcinomas (CSCCs) [34]. In addition, overexpression of NUCKS1 is strongly correlated with various characteristics of endometrial cancers (ECs), including FIGO stage, histologic grade, lymphovascular space involvement, lymph node metastasis, and recurrence, although NUCKS1 expression is not significantly correlated with other clinicopathologic factors [35]. Meanwhile, NUCKS1 has been identified as a candidate gene involved in distant metastasis in colorectal cancer (CRC) and gastric adenocarcinoma. However, there are no strong relationships between NUCKS1 and other clinical variables (such as gender and age) [36, 37]. Moreover, combined overexpression of NUCKS1 and Ki-67 (a well-known proliferation marker [38]) has been identified as an independent prognostic factor for both disease-free survival and overall survival in gastric adenocarcinoma patients [37]. In skin tumors, high expression of NUCKS1 in the nuclei of squamous cell carcinoma (SCC) and basal cell carcinoma (BCC) cells is more common than Ki67 expression. NUCKS1 expression has been found to be much lower in benign keratoacanthoma (KA) than in malignant tumors [39]. In breast carcinoma, NUCKS1 has exhibited higher expression than other investigated markers (including Ki67, estrogen receptor, progesterone receptor, human epidermal growth factor receptor 2, and cytokeratin 5/6) [40, 41]. Additionally, NUCKS1 is also highly expressed in breast cancer with obesity [42]; these findings are consistent with results obtained in a previous study [43]. NUCKS1 is a novel immunohistochemical marker for the early detection of hepatocellular carcinoma (HCC) [44]. Furthermore, NUCKS1 is elevated in human lung cancer (LC) tissues; this elevation is inversely correlated with miR-137 expression levels [45]. Moreover, NUCKS1 is one of the most significant transcription factors in LC and is predicted to be a therapeutic transcription factor [46]. Besides, after 4 Gy of total-body X-ray irradiation, Trp53+/− NUCKS1+/− mice developed tumors more rapidly than Trp53+/− mice [47]. In addition to cancer, NUCKS1 is highly expressed in tissues from human immunodeficiency virus- (HIV-) positive patients [48]. Intriguingly, NUCKS1 expression is elevated during the initial stages of embryonic development and then gradually decreases until birth in all tissues except for nervous tissue and muscle fibers [3]. Similarly, in in vitro studies, the results were also consistent with in vivo studies. NUCKS1 expression is increased in a highly invasive lung adenocarcinoma cell line [49]. Remarkably, NUCKS1 is critical for tumor suppression and the DNA damage response [50]. Taken together, the aforementioned findings indicate that NUCKS1 is highly expressed in many cancers and in HIV-positive patients and can be used as a marker for cancers. Furthermore, NUCKS1 is associated with the depth of invasion and tumor node metastasis classification, but there are no significant correlations between NUCKS1 expression and gender or age. In summary, further studies are needed to elucidate the mechanism by which NUCKS1 leads to oncogenesis. However, in contrast to the cancers mentioned above, in adult T-cell leukemia-lymphoma (ATLL), NUCKS1 expression has been found to be lower than the average NUCKS1 expression across all specimens [51]. In addition, NUCKS1 is reduced in acute kidney injury (AKI) [52], and the inhibition of NUCKS1 plays a role in cytokine modulation and facilitates corneal recovery following alkali burn [53]. Intriguingly, NUCKS1 knockout (NKO) mice [54] and hypothalamus-specific deletion of NUCKS1 (HNKO) mice [55] exhibit decreased insulin signaling, increased body weight/fat mass, impaired glucose tolerance, and reduced insulin sensitivity. Moreover, NUCKS1 has been shown to exhibit low expression in a mood disorder group relative to healthy controls (HCs) [56]. Recently, markedly lower NUCKS1 expression has been found in subjects with AIS compared with controls, particularly for patients with the TT genotype [32]. In addition to the in vivo studies mentioned above, the following in vitro studies showed the same results; in childhood acute lymphoblastic leukemia (ALL), compared with NUCKS1 expression in Reh cells overexpressing a scrambled control miRNA, NUCKS1 is downregulated in Reh cells overexpressing miR-125b, miR-99a, and/or miR-100 [57]. Notably, NUCKS1 knockdown sensitizes cells to mitomycin C and X-rays and promotes chromatid-type aberrations [50]. Overall, NUCKS1 is downregulated in lymphoma, acute inflammatory disease, metabolic diseases, mood disorders, and AIS (Figure 1).

## 4. Functions of NUCKS1 in Diseases

### 4.1. Metabolism

**Figure 2:**
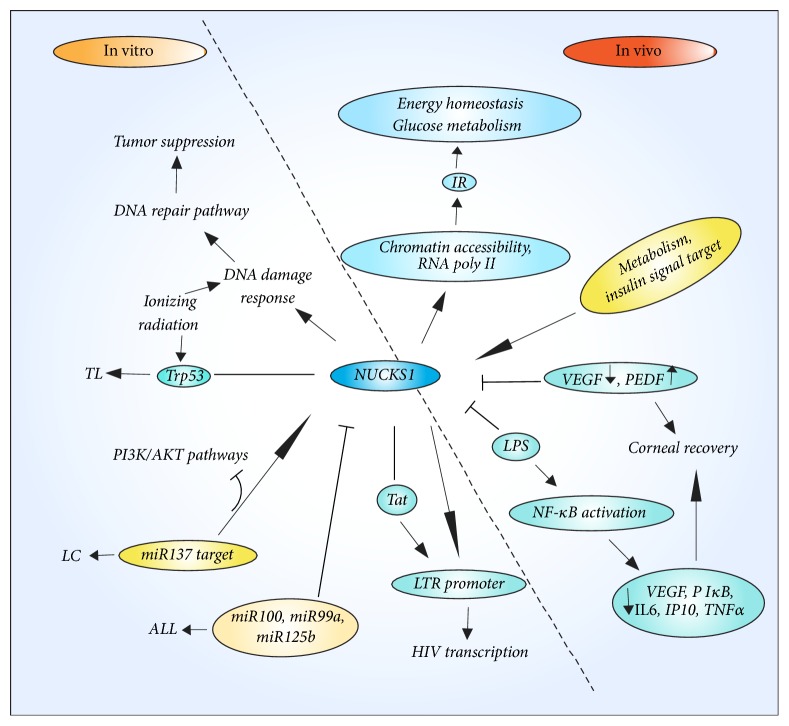
The metabolic pathway of NUCKS1 in physiological and pathological process. In in vivo studies, NUCKS1 plays the important roles in other metabolic diseases, especially involving in regulating insulin metabolism. NUCKS1 has correlation with the inflammatory immune diseases via involving in up-and-down certain cytokines and activating some pathways. In in vitro studies, NUCKS1 has connection with miRNA, but the expression level of them is inversely. Meanwhile, NUCKS1 plays significant roles in DNA damage response and participates in DNA repair pathway.

Notably, in in vivo studies, NUCKS1 has been found to be a transcriptional regulator of insulin signaling components, and genome-wide ChIP-seq has identified metabolism and insulin signaling as NUCKS1 targets [54]. Specifically, NUCKS1 is a physiological regulator of energy homeostasis and glucose metabolism that functions by regulating chromatin accessibility and the recruitment of RNA polymerase II (poly II) to promoters of insulin receptor (IR) and other insulin pathway modulators [54]. In addition, NUCKS1 has other targets that regulate insulin signaling, and it is common for transcription factors such as nuclear factor *κ*B (NF-*κ*B) or Myc to regulate multiple members of a signaling cascade to control physiological effects [58–61]. Furthermore, NUCKS1 can bind the promoter regions of a few genes in several pathways [54]. Hypothalamic NUCKS1 plays an essential role in regulating in vivo peripheral glucose homoeostasis and insulin signaling [55] (Figure 2).

### 4.2. Inflammatory Immune Response

In vivo studies results have suggested that the inhibition of NUCKS1 plays a role in cytokine modulation and facilitates corneal recovery [53]. Such inhibition decreases the angiogenic response and angiogenic vascular endothelial growth factor (VEGF) but increases antiangiogenic factors (such as pigment epithelium-derived factor (PEDF)) following in vivo alkali injury. Moreover, NUCKS1 regulates NF-*κ*B activation. Following lipopolysaccharide- (LPS-) induced NF-*κ*B activation, NUCKS1 knockout corneal epithelial cells showed reduced expression of phosphorylated I*κ*B (P I*κ*B), interleukins (including IL6), IP10, and tumor necrosis factor *α* (TNF*α*). Given the aforementioned data, NUCKS1 regulates NF-*κ*B, the expression of particular NF-*κ*B-mediated cytokines, and the phosphorylation of specific proteins critical to the NF-*κ*B signaling pathway in ocular epithelial cells [53]. Remarkably, in in vitro study, NUCKS1 has been shown to increase Tat-mediated transcriptional activity of the HIV-1 long terminal repeat (LTR) in an NF-*κ*B-independent manner [48] (Figure 2).

### 4.3. NUCK1 and MicroRNA

NUCKS1 directly interacts with miR-92a-3p and miR-29b-3p in BD patients [62]. Meanwhile, in in vitro studies, NUCKS1 is downregulated upon enforced expression of miR-125b in combination with miR-99a and/or miR-100 compared with scrambled control-miR in ALL [57]. In addition, miR-137 3′-UTR directly targets NUCKS1 by binding to its seed region in LC cells. Furthermore, miR-137 inhibits PI3K/AKT pathways by targeting NUCKS1 [45]. In addition, siRNA silencing of NUCKS1 significantly suppresses tumor growth. The inhibition of NUCKS1 affects changes in the expression of proteins involved in cell signaling, nucleic acid metabolism, and several microRNAs (including miR4796, miR1305, miR4762, and miR4445) [44] (Figure 2).

### 4.4. DNA Damage Repair

NUCKS1 resembles other members of the HMG family, which dominate chromatin remodeling and regulate gene transcription. It has been reported that Trp53+/− NUCKS1+/− mice frequently succumbed to CD4− CD8− thymic lymphomas (TLs). In the context of Trp53 deficiency, wild-type levels of NUCKS1 are required to suppress radiation-induced TLs; this suppression likely involves the role of the NUCKS1 protein in the DNA damage response [47]. Recently, in in vitro studies, NUCKS1 is a novel RAD51 associated protein 1 (RAD51AP1) paralog that is important for homologous recombination (HR) and genome stability [50]. Moreover, NUCKS1 is a chromatin-associated protein with a role in the DNA damage response and in HR, a DNA repair pathway critical for tumor suppression [50]. However, at the core of the DNA damage signaling apparatus is a pair of related protein kinases, ataxia telangiectasia mutated (ATM), and ATM and Rad3-related (ATR) that are activated by DNA damage [63]. Notably, NUCKS1 is a substrate of either ATM or ATR following exposure to ionizing radiation [64]. Intriguingly, NUCKS1, which has been confirmed to be associated with DNA replication, is highly expressed in S-phase cells during DNA damage repair following ionizing radiation [50] (Figure 2).

## 5. Conclusions and Perspectives

GWAS have clarified that NUCKS1 is a susceptibility gene for various diseases. Furthermore, GWAS have also shown that a single disease can be associated with multiple SNPs of NUCKS1. However, NUCKS1 has diverse expression in different races, even when the same SNP of NUCKS1 and the same disease are examined. Moreover, NUCKS1 genotypes exhibit distinct expression for certain diseases. The exact roles of NUCKS1 in diseases remain obscure, although it has been reported that expression and transcription levels of NUCKS1 are significantly associated with PD [13, 22] and that NUCKS1 transcription and methylation are likely to play a vital role in youths with BD [30]. In addition, tumors with high NUCKS1 expression have exhibited increased invasive potential [35, 40]. NUCKS1 may promote tumor cell proliferation, progression, and invasion [35, 36, 44, 49]. Similarly, NUCKS1 may have a role in signal transduction in cancer [44]. Furthermore, NUCKS1 could be an independent prognostic factor for cancer recurrence [34, 36]. Notably, NUCKS1 plays a vital role in mitigating replication stress in human cells. NUCKS1 expression may be upregulated as part of the activated DNA damage response network during tumorigenesis. In addition, NUCKS1 may be associated with a selective advantage during tumor initiation and development that allows early neoplastic cells to overcome replication stress [47]. However, the relationship between elevated levels of NUCKS1 and tumor progression is currently unknown, and further work is needed to clarify the roles of NUCKS1 in tumor progression. In contrast, NUCKS1 has low expression in acute inflammatory diseases. This finding may be associated with the role of NUCKS1 as a transcription factor to regulate NF*κ*B activation and release NF*κ*B-mediated cytokines [53]. Furthermore, NUCKS1 is also downregulated in metabolic diseases [54, 55]. It is highly likely that modifications in NUCKS1 could link insulin signaling and energy homeostasis to changes in myriad cellular processes [54]. Future studies directed at understanding the tissue- and stimuli-specific downstream targets of NUCKS1 may provide promising therapeutic approaches in the treatment of obesity and insulin resistance [54]. Moreover, neuron-specific depletion of NUCKS1 will provide a better understanding of metabolic phenotypes [55]. Such understanding will provide guidance for exploiting NUCKS1 as a potential target in the treatment of impaired glucose homoeostasis [55]. Similarly, NUCKS1 has been found to be decreased in a mood disorder group relative to a HC group. This observation may be correlated with transcriptional regulation by NUCKS1 via chromatin remodeling [5]. In addition, in AIS, rs951366 maps the 3′ untranslated region of NUCKS1, which encodes a cell cycle-related protein and plays an important role in cell proliferation and cell progression. In addition, the LD block of rs951366 maps a DNase I hypersensitivity site in multiple cell lines and might also be located in a strong enhancer region marked by several active histone methylation modifications [65]. The rs951366 locus has the potential to regulate the transcriptional activity of NUCKS1 and therefore merits additional functional analysis. Overall, the functions of NUCKS1 have remained largely unknown, and more studies are needed to reveal more details about these functions in the future.
